# Self-Medication Practices among the Adolescent Population of South Karnataka, India

**DOI:** 10.1155/2020/9021819

**Published:** 2020-09-07

**Authors:** Edlin Glane Mathias, Anjalin D'souza, Savitha Prabhu

**Affiliations:** ^1^Manipal College of Nursing, Manipal Academy of Higher Education (MAHE), Manipal, Karnataka, India; ^2^Department of Child Health Nursing, Manipal College of Nursing, Manipal Academy of Higher Education (MAHE), Manipal, Karnataka, India; ^3^Department of Mental Health Nursing, Manipal College of Nursing, Manipal Academy of Higher Education (MAHE), Manipal, Karnataka, India

## Abstract

**Introduction:**

Self-medication is used every day in the form of self-care of our health. Different studies in India have shown that more than 50% of the adolescent population takes self-medication every day for their health. Self-medication is an important concern at the global level, and it is an important issue in the health area.

**Aims:**

To determine the prevalence of self-medication among adolescents, identifying the commonly used drugs as self-medication and finding the association between self-medication and selected demographic variables are important.

**Materials and Methods:**

In a descriptive cross-sectional survey, 220 adolescents were enrolled through cluster random sampling. A self-administered questionnaire (developed by the researcher) along with a demographic profile sheet to assess the prevalence of self-medication and commonly used drugs was exercised. In the study, self-medication was defined as the consumption of medication without the prescription of the physician in the past year. Data analysis was performed by descriptive and inferential statistics using SPSS 16.0 software, and the significance of *p* value (<0.05) was considered.

**Results:**

Around 120 (54.5%) adolescents were in the age group of 17 years; among them, 123 (55.9%) were females. The prevalence of self-medication was found to be 173 (78.6%). Antipyretics were consumed by 147 (78.6%) adolescents and antitussives by 120 (54.5%). It was observed that 110 (50%) of the adolescents preferred allopathic system of medication. It was also reported that 52 (23.6%) adolescents self-medicated continuously (i.e., for a month). The results also showed that factors like the type of family (*χ*^2^ = 9.615, *p* < 0.05), father's education (*χ*^2^ = 13.791, *p* < 0.05), mother's education (*χ*^2^ = 14.633, *p* < 0.05), and distance from a nearest medical store (*χ*^2^ = 17.290, *p* < 0.05) were associated with self-medication.

**Conclusion:**

The present study has shown that the prevalence of self-medication is high among adolescents, and most of them had taken it without consulting a doctor. The study concludes that it is important to create awareness among adolescents regarding self-medication.

## 1. Introduction

Self-medication practice (SMP), an element of self-care, is the consumption of medication without being prescribed by the healthcare professionals (e.g., resubmitting old prescriptions, sharing medication with relatives/family members, or using leftover medications) for the treatment of self-recognized illnesses [1]. Concerns about the practice of self-medication (SM) are based on associated risks such as adverse drug reactions, disease masking, inaccurate diagnosis of disease, increased morbidity, drug interactions, wastage of healthcare resources, and antibiotic resistance. The World Health Organization (WHO) has defined self-medication as the use of drugs to treat self-diagnosed disorders or symptoms, or the intermittent or continued use of prescribed drugs for chronic or recurrent disease or symptoms [2]. Self-medication thus forms a significant vital portion of self-care, which can be defined as the chief public health resource in the healthcare system. It includes self-medication, nondrug self-treatment, social support in illness, and first aid in every day life. According to William Osler, a great feature which distinguishes man from animals is the desire to take medicine [3]. In 1995, the WHO expert committee on National Drug Policies (NDP) stated “self-medication is widely practiced in both developed and developing countries. Medications may be approved as being safe for self-medication by the national drug regulatory authority. Such medicines are normally used for the prevention or treatment of minor ailments or symptoms, which do not justify medical consultation. In some chronic or recurring illnesses, after initial diagnosis and prescription, self-medication is possible with the doctor retaining an advisory role” [4]. Not much is known about health-related problems and healthcare utilization, including self-medication among young adults. The youth are highly influenced by the media and the Internet, which promotes self-medication behavior. The increased advertising of pharmaceuticals poses a larger threat of self-medication to the younger population in general. This raises concerns about incorrect self-diagnosis, drug interaction, and the use of drugs other than for the original indication. The increase in the quantities and varieties of pharmaceuticals worldwide eases the accessibility of medicine by consumers, thereby giving options for its misuse. A study from Nigeria has observed self-medication as a common practice among a group of health workers that included dental, midwifery, and nursing students. It has been suggested that self-prescription is also prevalent among practicing physicians [5].

Through the literature review, it is clear that most of the adolescents choose self-medication. The researcher identified that there are limited studies that have been conducted on preuniversity college adolescents among South Karnataka. Hence, there was a need to recognize the prevalence of self-medication among adolescents and identify the commonly used drugs and the factors associated with self-medication, which helps the health professionals to focus the attitude of adolescents towards self-medication and take appropriate actions.

## 2. Materials and Methods

A descriptive cross-sectional survey was conducted among 220 adolescents studying in three different preuniversity colleges of Udupi District, Karnataka, in January 2016 (one month). The colleges had different streams of subjects such as science, commerce, and arts. Participants were selected based on cluster sampling (the entire class was taken as a cluster).

The sample size was calculated based on the pilot study findings, where relative precision was kept 10% of proportion, and anticipated precision was 0.83%; cluster effect of two was applied. The approximate sample size taken was 220. Ethical permission was obtained (IEC 680/2015) for conducting the study. Formal written permission from the college authority, written consent from the parents, and assent from the participants were obtained before the study. The purpose of the study was explained to the participants, and the confidentiality of the information was maintained. The researcher presented questionnaires to the participants, and they took thirty minutes to complete the task. Data collected were entered and statistically analyzed using the Statistical Package for Social Sciences (SPSS) version 16.0. The sample characteristics, prevalence, and associated factors were described using frequency and percentage. A chi-square test was used to find the association between the prevalence of self-medication and demographic variables.

### 2.1. Sample Size

The sample size was calculated using the following formula:

(1) $$\begin{array}{c} n=\frac{{\left(Z_{\left(1-\alpha /2\right)}\right)}^{2}pq}{{\left(€p\right)}^{2}}, \end{array}$$

where *n* = minimum sample size required, *p* = anticipated prevalence of self-medication, *q*=1 − *p*, € = relative precision, €*p* = margin of error, *p* = 0.83 (83%), *q*=1 − *p* (1–0.83 = 0.17), and € = 10% of *p*. For cluster sampling, sampling size × design effect =78.67 × 2 =157.34.

### 2.2. Description of the Tool

Tools used for the study were demographic pro forma and self-medication assessment tool. These tools were developed by the researchers. The demographic tool consisted of 12 items, and the self-medication assessment tool consisted of 26 items. The tools were validated by seven experts, and the reliability of the tool in English was assessed using the test-retest method and was found to be 0.8. The tool was translated to Kannada because the medium of education for some colleges was in the Kannada language; the reliability of the tool in Kannada was found to be 0.8.

### 2.3. Statistical Analysis

Data collected were entered and statistically analyzed using the Statistical Package for Social Sciences (SPSS), version 16.0. The sample characteristic, prevalence, and associated factors were described by using frequency and percentage. A chi-square test was used to find the association between the prevalence of self-medication and demographic variables.

## 3. Results

### 3.1. Distribution Based on Sociodemographic Variables (*N*: 220)

The total number of adolescents selected for the study was 220. In this study, 173 (78.6%) adolescents were found to be practicing self-medication, out of which 123 (55.9%) of them were females and 147 (66.8%) were males. The majority of the adolescents (197 (89.5%)) belonged to the rural area, 142 (64.5%) belonged to the nuclear family, and 175 (79.5%) adolescents reported that the distance from the nearest medical store was 1–4 km (Table 1).

### 3.2. Distribution of Commonly Used Drugs as Self-Medication (*N*: 173)

**Figure 1:**
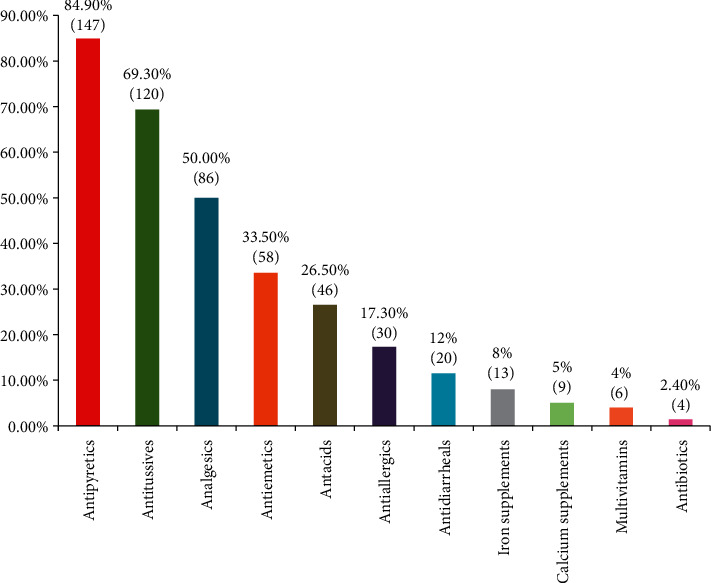
Commonly used drugs as self-medication (*N*: 173).

The prevalence of self-medication was found to be 78.6%. The adolescents who had taken self-medication were further analyzed for specific drugs they had taken. Most of the adolescents took antipyretics (147 (66.8%)), followed by antitussives (120 (54.5%)) and lastly analgesics (86 (39%)) (Figure 1).

### 3.3. Distribution of Practices of Using Self-Medication (*N*: 173)

It was also reported that, among 173 adolescents, 110 (63.5%) adolescents preferred the allopathic system of medicine, and 110 (63.5%) preferred tablets. It was surprising that 52 (30%) adolescents had taken self-medication for a month. Among 173 adolescents, 52 (30.0%) had checked the expiry dates at the time of purchase, and the adolescents remembered the brand name while buying the drug. Among the 173 adolescents, 81 of them (46.9%) consulted a physician while treating the illness (Table 2).

### 3.4. Distribution of Adverse Effects due to Self-Medication (*N*: 24)

Adolescents also experienced few adverse effects due to self-medication; 9 (5.2%) of them reported headache, 2 (2.8%) allergy, and 4 (2.3%) diarrhea (Table 2).

### 3.5. Distribution of Drugs Stored at Home (*N*: 162)

Commonly stored drugs at home were as follows: Tab. Calpol 44 (27.3%) and Panadol 36 (22.4%) and syrup Benadryl 15 (9.2%) (Table 2).

### 3.6. Distribution of Reason for Using Self-Medication (*N*: 173)

The majority of adolescents (102 (59.2%)) reported the reason for taking self-medication was severe illness, and at that moment, they were not able to visit any doctor; 35 (20.2%) had self-medicated because of their busy schedule, and 7 (4.0%) had referred the previous prescription. Most of the adolescents (89 (51.7%)) also informed that the source of information was parents and 42 (24.2%) from mass media (Table 3).

### 3.7. Distribution of Association between Self-Medication and Selected Variables (*N*: 173)

The study further revealed that there is a significant association between the use of self-medication and the family background (*χ*^2^ = 9.615, *p* < 0.05), father's education (*χ*^2^ = 13.791, *p* < 0.05), and mother's education (*χ*^2^ = 14.633, *p* < 0.05) (Table 4).

### 3.8. Other Findings

Antipyretics were consumed by 147 adolescents, and the reason stated by 86 (58.5%) was emergency, 7 (4.76) minor illness, and 35 (23.8%) hectic schedule; 6 (4%) stated that doctor consultation fee is expensive, 6 (4%) symptoms were severe, 3 (2%) state that clinic is far away, and 2 (1.36%) do not remember the reason, but the maximum days taken were 1–3 days; most of the adolescents (38 (25.8%)) had consumed Tab. Calpol.

Antitussives were utilized by 120 (69.30%) adolescents, and the reasons stated were emergencies by 56 (46.6%), parental advice by 29 (24.2%), and hectic schedule by 35 (29.1%). The maximum days they self-medicated were 1–5 days, and most of the adolescents had utilized syrup Benadryl (25.8%).

Analgesics were taken by 86 (50%) adolescents, and the reason stated was emergency by 79 (91.8%) and parental advice by 7 (8.1%). They took it for a maximum of 1–4 days, and most of the adolescents took tablet Combiflam.

It was surprising to find that 58 (33.5%) adolescents had taken antiemetics, and the reason stated was emergency by 29 (50.3%), busy schedule by 9 (15.5%), teacher's advice by 6 (10.3%), and difficulty in getting doctor appointment by 6 (10.3%), and 8 (13.7%) reported the distance of pharmacy being far away. The study further revealed that most of the adolescents (48 (82.7%)) had taken antiemetics for just 1 day, and the drug was Tab. Domstal 25 (43.1%). It was observed that 46 (26.50%) adolescents had taken antacids, 30 (17.30%) antiallergics, 20 (12%) antidiarrheals, and 4 (2.4%) had taken antibiotics without consulting any doctor. Antacids and antiallergics were consumed for 1–3 days while antibiotics for a month by two adolescents (tablet cefixime 200 mg).

## 4. Discussion

### 4.1. Prevalence of Self-Medication

The findings of this study show that, among 220 adolescents, the majority of the adolescents (197 (89.5%)) belonged to the rural area. 173 (78.6%) adolescents were found to be practicing self-medication; out of which, 123 (55.9%) of them were females and 147 (66.8%) were males. A study in Puducherry showed the prevalence was as high as 71% [3]. A study in urban Delhi showed that the prevalence of self-medication among those who had suffered some illness episodes in the past 1 month was 31.3% [6]. Another study in an urban slum showed that the self-medication was practiced by 34.5% of respondents and prevalent among all the age groups [7]. A recent study from Sri Lanka had reported 12.2% and 7.9% prevalence of self-medication to allopathic drugs from an urban and rural area, respectively, two weeks prior to the interviews [8]. The study from South Africa had shown a very high prevalence of self-medication (93–98%) [9]. However, all these studies had taken accountable types of drugs including homeopathy or other Indian system-related drugs. The studies on self-medication practices among medical students in Accra, Ghana, showed higher prevalence rates [10].

### 4.2. Commonly Used Drugs for Self-Medication

The present study shows that most of the adolescents (147 (66.8%)) took antipyretics, followed by antitussives (120 (54.5%)) and analgesics (86 (39%)). 17 (44.7%) out of the 38 users of antibiotics got hold of it from their respective homes and other sources. This is in contrast with many studies that reported the major sources were pharmacies, patent medicine stores, friends, and families [11]. Paracetamol (75.1%) was the most used drug among the students. Similar reports were found in other studies as well [12]. Tetracycline (34.2%), amoxicillin (28.9%), and metronidazole (18.4%) were the most used antibiotics. Studies also reported that ampicillin and amoxicillin were the most self-medicated drugs among students [13]. One-third (33%) of the students reported using antimalarial drugs without prescription. Artemether/lumefantrine (37%), artesunate (21%), and sulfadoxine + pyrimethamine (16%) were the most commonly used antimalarial drugs. Self-medicated antimalarial use was also reported to be prevalent in some studies among tertiary institution students [14]. Self-medication practices with antibiotics in some studies were mostly reported to be for urinary tract infection, sore throat, gastrointestinal ailments, and cough [15].

### 4.3. Factors Associated with/Promoting Self-Medication

This study also portrays that the adolescents also experienced few adverse effects due to self-medication such as headache reported by 9 (5.2%); 2 (2.8%) reported allergy, and 4 (2.3%) reported diarrhea. This finding was supported by studies conducted in Meket, Ethiopia, China, southern India, and Nigeria. The possible justification might be due to the inability of the participants to afford healthcare fees and lack of time to consult healthcare professionals. Therefore, improving the perception of participants about the quality of healthcare services, creating awareness, and managing peer pressure may reduce self-medication practices [16]. A study in Porto Alegre/RS (Southern Brazil) showed that almost 80% of those who practiced self-medication was influenced by laypeople, with the vast majority being recommended by friends or family members, with the self-medication being an occasional use. There are indications that self-medication is associated with stocks of medicine at home, which can facilitate this risky behavior of self-medication for health-related problems [17].

## 5. Limitation

In this study, the analysis was based on self-analysis with the possibility of over- or underreporting. With the questionnaire provided by the researcher, only limited information was captured.

## 6. Conclusion

In conclusion, our findings demonstrated that the prevalence of self-medication was high among adolescents. Most of the drugs were stored at home and consumed by referring to previous prescriptions. It is important to educate the public and provide counseling to adolescents regarding the risks of taking self-medication.

## Figures and Tables

**Table 1:** Distribution based on sociodemographic variables (*N*: 220).

Sample characteristics	Frequency (*f*)	Percentage (%)
Age in years		
16	62	28.2
17	120	54.5
18	38	17.3

Gender		
Male	97	44.1
Female	123	55.9

Area of living		
Rural	197	89.5
Urban	23	10.5

Type of family		
Extended	17	7.7
Joint	61	27.8
Nuclear	142	64.5

Recently suffered from health problems		
Yes	99	45.0
No	121	55.0

Education of father		
No formal education	8	3.6
Primary	95	43.2
High school	69	31.3
Preuniversity	34	15.6
Graduate	14	6.3
Postgraduate	0	0

Education of mother		
No formal education	0	0
Primary	96	43.6
High school	79	35.9
Preuniversity	32	14.6
Graduate	11	5.0
Postgraduate	2	.9

Father's occupation		
Nonprofessional	56	25.5
Professional	25	11.4
Semiprofessional	139	63.1

Mother's occupation		
Nonprofessional	161	73.6
Professional	16	7.3
Semiprofessional	43	19.1

Distance from the nearest medical store in km		
1–4	175	79.5
5–8	25	11.5
Do not know	20	9.0

Extended family: family which resides with grandparents; joint family: a type of family composed of parents, children, their spouses, and offspring in one household; nonprofessional: describes someone who does something not as their job but because they are interested in it; professional: a member of a profession or any person who earns living from a specified professional activity; semiprofessional: an occupation that requires advanced knowledge and skills but is not widely regarded as a true profession.

**Table 2:** Distribution of practices of self-medication (*N*: 173).

Sample characteristics	Frequency (*f*)	Percentage (%)
Treated continuously with self-medication (*n* = 52)		
Yes	52	30.0

Duration of treating with self-medication (*n* = 52)		
1–4 days	17	9.8
5–8 days	35	20.3

Expiry date verification		
Yes	52	30.0
No	121	70.0

Verification of expiry dates (*n* = 52)		
After consumption	12	23.0
Before taking	31	59.7
Forgot	2	3.84
Time of purchase	7	13.5

Reading instructions in the medicine package		
Always	101	58.5
Sometimes	70	40.4
Never	2	1.1

Adverse events (*n* = 24)		
Allergy	5	2.8
Diarrhea	4	2.3
Headache	9	5.2
Rashes	2	1.1
Vomiting	4	2.3

Medications stored at home (*n* = 162)		
Syrup Alex	11	6.7
Syrup Benadryl	15	9.2
T. Paracetamol	44	27.3
T. Panadol	36	22.4
T. Grenil	2	1.2
T. Combiflam	6	3.7
T. Metacin	4	2.4
Do not remember	44	27.3

**Table 3:** Distribution of reason for using self-medication (*N*: 173).

Sample characteristics	Frequency (*f*)	Percentage (%)
Reasons for taking self-medication		
Hectic schedule	35	20.2
Doctor consultation is costly	6	3.4
Distance of clinic is far	11	6.4
Emergency conditions	102	59.2
Difficulty in controlling the symptoms	3	1.7
Minor illness	7	4.0
Advice of mother/parent	7	4.0
Do not remember	2	1.1

Source of information		
Mass media/social network	42	24.2
Friends	11	6.3
Parents	89	51.8
Pharmacist	17	9.8
Previous prescription	7	4.0
Books	5	2.8
Do not remember	2	1.1

Siblings advice for self-medication		
Yes	58	33.5
No	115	66.5

**Table 4:** Distribution of association between self-medication and selected variables (*N*: 173).

Variables	Use of self-medication				
	Using (*f*)	Not using (*f*)	*χ* ^2^	d*f*	*p* value
Age in years					
16	50	12	1.413	2	0.493
17	91	29			
18	32	6			

Gender					
Male	80	17	1.514	1	0.143
Female	93	30			

Area of living					
Rural	153	40	1.172	1	0.230
Urban	20	7			

Type of family					
Extended	17	7	9.615	2	^*∗*^0.008
Joint	44	10			
Nuclear	112	30			

Education of father					
No formal education	8	6	13.791	5	^*∗*^0.017
Primary	76	10			
High school	48	17			
Preuniversity	27	8			
Graduate	14	6			

Education of mother					
Primary	74	15	14.633	4	^*∗*^0.005
High school	56	10			
Preuniversity	30	8			
Graduate	8	6			
Postgraduate	5	8			

^*∗*^Significant at 0.05 level.

## Data Availability

The data used to support the findings of this study are available from the corresponding author upon request.
